# Reframing the role of communication in consensual and/or ethical (non)monogamies: A proposal for a change in academic terminology

**DOI:** 10.12688/openreseurope.17502.1

**Published:** 2024-08-05

**Authors:** Daniel Cardoso

**Affiliations:** 1 CICANT, Lusófona University of Humanities and Technologies, Lisbon, Lisbon, Portugal; 2 FCSH, NOVA University of Lisbon, Lisbon, Portugal

**Keywords:** consensual non-monogamy; ethical non-monogamy; taxonomy; onomastics; hypernyms; meta-methodology

## Abstract

In this essay, I focus on the politics and impacts of naming, especially in the social and human sciences, and more specifically on studies that focus on subordinated or discriminated groups. Through this essay, I argue that naming conventions are some of the most important – and dangerous – tools and acts that researchers have at their disposal and, thus, should be employed with the utmost care.

Considering the ongoing discussions – both inside and outside of academia – around the terms “consensual non-monogamies” and “ethical non-monogamies”, this essay proposes a novel solution to help create less morally-slanted, and overreaching, hypernyms, or umbrella terms.

Here, I provide a rationale for using “explicitness” as a fundamental concept on which to build new classificatory hypernymic terminology. The terminology proposed is equally applicable (and applied) to both monogamies and non-monogamies, and is tripartite: Explicit, Implicit, and Covert.

In addition to the terminological proposal, I include in this essay a series of intended limitations and constraints to its usage, its interoperability with other systems, objections to (and responses to) the usage of this system, and advantages of this novel classificatory system, as well as an outline of how this proposal might be improved upon.

## Introduction – On naming

Conceptual accuracy is fundamental to research, but always relies on epistemological and disciplinary commitments to what constitutes ‘enough’ specificity and what the object of that specificity should be. The importance of this can be illustrated by considering the central role of taxonomy in science, and how taxonomical changes or attributions can be so hotly contested – and how they necessarily resort to boundary work. Discussions of the Anthropocene exemplify this: the Anthropocene, its definition, its boundary conditions, and the multi-year process that surrounded the various proposals for its definition ( Malhi, 2017), alongside the ‘hope’ that plastic would be a good objective boundary marker ( Chen *et al*., 2022) and how that hope has been recently dashed ( Dimante-Deimantovica *et al*., 2024), are all important reminders of the complexity of naming, of how naming never seamlessly arises out of mere observation of a supposedly fixed reality.

While the fundamental importance of conceptual accuracy is true for geology and other ‘hard sciences’, it is also so for the Social and Human Sciences, where the words and terminology that people use and have available can have an enormous impact on their and others’ lives. The struggles over naming (oneself or one’s group), labelling ( Becker, 1997), self-representation, and many other such terms have been studied over and over again – especially when it comes to socially-minoritized groups and terms (e.g., “queer”, “gay”, “slut”).

Valerie Alia (2007, p. 458) puts it succinctly and brilliantly: “naming is inseparable from other political phenomena and is an important key to understanding power relations in a range of settings”; she calls it “ *political onomastics*, the politics of naming”.

Taken to the ultimate degree, we need to echo Gayatri Spivak’s rhetorical question about whether the subaltern can speak ( Spivak, 1994), and what the subaltern is allowed to say or not. We need to consider the historical systems that result in epistemic inequalities around naming and defining, and who gets to speak truth to the world, and about the world ( Fricker, 2007). We need to consider the situated experience ( Haraway, 1988) that is producing knowledge – and that naming is a powerfully symbolic form of that. Readers from a Western, Christian-backed tradition are certainly familiar with the symbolism of naming (non-human) animals ( Borkfelt, 2011), given that Adam’s task of naming all living things was both a divine mandate and a symbol of humankind’s dominion over the rest of the world – there is power in naming, and naming is power.

Two major theoretical connections that pertain to naming have to do with, on the one hand, identity (which we can conceptualize in both sociological and psychological terms ( Bamberg, 2011; Butler, 2005; Hall, 2006; Hammack, 2008)), and, on the other hand, recognition ( Clohesy, 2013; Egan & Hawkes, 2009; Hines, 2013; Honneth & Rancière, 2016). From both lines of inquiry (identity and recognition) we can easily see how fundamental these processes – these politics! – of naming are to Self and Other, to both the individual and the sociopolitical levels.

Keeping in line with my own central theoretical background, however, I would like to shift the focus to a Foucauldian approach to subjectivity – or, more specifically, to subjectification ( Foucault, 2000a; Foucault, 2002; Foucault, 2013; Foucault, 2017) and to *assujetissement*. Foucault explores at length how the production of subjectivities relies on the production of rules of subjectivity, to which someone becomes subjected to, to be a subject *of*.

As many authors have noted, these terms illuminate subjecthood as the result of an *ambivalent process*, historically and culturally situated ( Cornu, 2014). The focus here is not so much on the processes or modes of subjectification, but rather on its ambivalent nature – because naming (the Self or the Other) requires the deployment of a normative system. To be a subject *of*, one must be subject(ed) *to* – a somewhat simplistic explanation of how subordinated groups produce their own disciplinary practices and normative systems (see de Oliveira, 2013, for a more detailed description). This also helps us understand the paradigmatic difference between queer-as-verb and queer-as-noun ( Halperin, 2003; Sullivan, 2003), as verbs don’t constitute identitary nexuses the way nouns do. These systems do not, evidently, arise *ex nihilo*, rather they are shaped by existing ideologies and discourses.

We are back, then, to boundary work; to the line ( Rubin, 2007) between the acceptable and the unacceptable, and the constant negotiation between autonomy and the disciplinarity of becoming-subject. Even in other philosophical traditions, it is clear that there is a doing in the naming of things, and in the using of some terms but not others, that has consequences on ourselves and others around us ( Burgess & Plunkett, 2013). As I have delineated some of the stakes of these boundarizing and disciplining projects of naming, these provide a basis for the importance of umbrella terms, which I now turn to.

## On umbrella terms as an amplification of onomastic politics

If naming is as important and salient as shown above, then the creation of hypernyms – or umbrella terms, as they are more commonly known – is doubly so. Doubly because, on the one hand, there is the *naming* of the hypernym and, on the other, the conjoining and disjoining of a multitude of phenomena, objects, and subjectivities. Several of the examples above show how naming often occurs through hierarchical systems (e.g., a taxonomy has multiple layers of aggregation).

Whenever hypernyms are involved, and even if they do not necessarily, in some cases, involve hierarchical comparisons or systems of meaning, there is always a component of articulating why different names (i.e., ‘things’) can or should or might be understood together. The togetherness of those ‘things’ is always produced by whatever analytical system creates the hypernym, on top of the naming system underlying the hypernymic aggregation. As names, subjectivities and identities are (almost) always hypernymically connected, they (almost) always exist simultaneously as themselves and as part of something else (for example, ‘woman’ also exists as ‘gender’; ‘gay’ also exists as ‘sexual orientation’).

The importance, complexities, and possibilities that hypernyms offer (and fail to offer) in the context of taxonomies are central to one of the earliest known texts to look at the history of taxonomy, back in 1971 – and already back then, it was the promises of “modern electronic data processing equipment” ( Raven *et al*., 1971, p. 1213) and its ability to constantly rework data into *different* taxonomical arrangements that was seen as a boon. Such a hope is telling: the authors themselves consider pointless any totalizing project of data collection, focusing instead on the easy reworking of said data into (taxonomical) configurations that do not reveal an underlying true nature of the world, but rather allow for easier mobilization of the existing data in the context of specific purposes. One of the points its authors deemed as “high priority” was “the search for original ways of looking at the structure of nature” ( Raven *et al*., 1971, p. 1213). While they were strictly talking about biological taxonomies, I argue that the principle holds for any taxonomic system – because it is about the nature of taxonomies, not of biology (or any other science).

This is yet another clear example of what Foucault (1994a) calls power-knowledge – the intertwining of knowing *about* as a condition and a legitimation for acting *on*. It is not simply that knowing something allows for the manipulation of it, but rather that the creation of entire areas of knowledge brings into being a series of phenomena that then become open to being acted upon, in certain ways – the most obvious example here being that the *creation* of a category called ‘sexuality’ allowed for its objectivation and objectification, and thus for interventions over and on it.

In this sense, and considering the previous section, the ambivalent nature of *assujetissement*, or the way the moral lines between normality and abnormality are drawn and policed, are never piecemeal. Certain subjectivities are seen as being always in articulation (e.g., ‘man’ and ‘woman’), but they are in articulation within a certain framework that ascribes characteristics – taxonomic variables – underlying those subjectivities, a framework that makes those subjectivities visible as being in relation to one another.

Contested though the specific rules or characteristics of each of those subjectivities may be, if the contestation takes place within a single framework, it will reinforce the (normative) operations of power-knowledge involved in the birthing of that framework. Foucault (2000a) aptly noted the importance of not relying on any notion of gay (read, queer) culture as being inherently liberatory, since it brings with itself the risk and potential for siloing off huge swaths of cultural expression.

A fight for sexual liberation articulated strictly within the confines of ‘sexuality’ (even of a putative expanded definition of sexuality) is a self-defeating proposition ( Foucault, 1994a). As Schudson and van Anders note (2019, p. 357), “the emergence of new gender and sexual identity labels can sometimes produce more ways for individuals to define sharp boundaries”, and those boundaries then get deployed into disciplinary and normative dynamics of policing identities, behaviors and bodies. It is no wonder that some of the most radical proposals in the area of gender and sexualities strike precisely here – aiming for an excess of knowledge, terms and classifications that renders entire systems of subjectivity (e.g., gender) practically useless ( Preciado, 2011), and thus dysfunctional from a power-knowledge perspective. In a way, this is precisely what Raven *et al*. were cautioning against – a glut for information might make taxonomies *harder* to mobilize, not easier.

Lorde (2018) put it more pointedly: “the master’s tools will never dismantle the master’s house”.

Hypernyms are, then, an example of these tools *par excellence*. As noted above, the cojoining and disjoining of certain elements or characteristics brings into being entire sets of purportedly co-constitutive subjectivities, as well as how they are knowable as such and, so, what counts as true (and/or relevant) knowledge. If the politics of naming facilitate morally hierarchical relations between different subject positions, the politics of hypernyms – as inscribed in certain contexts of power-knowledge – create and constrain even the possibilities of *types* of subject positions that are intelligible in a given time and space. Those politics can, furthermore, reorganize the relations between certain sets of subject positions in ways that reify extant (or upcoming) configurations of power-knowledge.

They also constitute a potential for what Katharine Jenkins (2023, p. 71) calls “ontic injustice”, i.e., being “socially constructed as a member of a certain social kind where that construction consists, at least in part, of their falling under a set of social constraints and enablements that is wrongful to them”; and its subset, “ontic oppression”. This type of injustice and oppression does not require that harm is suffered by someone or some group, but stems from the “social kind” (her terminology for a certain socially-construed identity) that one is seen as being,

Because hypernyms are, in a way, one step above the subjectivities they organize under and inside them (and thus become conditions for the possibility of those same subjectivities), they can become harder to resist, and their impacts and effects can be far-reaching. Case in point, the way the coming into being of ‘sexuality’ had, as a precondition, the uncoupling of certain events, features and acts from the wider hypernym of ‘madness’ ( Foucault, 2006), to then become itself a hypernym (e.g., child sexuality, adult sexuality, deviant sexuality) and to spawn other hypernyms (e.g., sexual orientation, paraphilia).

Following this, I will now look at one specific set of hypernyms related to monogamy, and attempt to trace the layers surrounding them.

## Storying oppositions to monogamy and their namings

Various Global North authors – contemporary and not – have argued against, and/or critically analyzed, monogamy and its hegemonic position within the field of gender and sexualities (i.e., mononormativity) ( Carter, 2006; Engels, 2010; Pieper & Bauer, 2005; Pieper & Bauer, 2014; Porto, 2018; Rosa, 1994; Rubin, 2007; Ryan & Jethá, 2010). An actually representative list would be enormous and besides the point – I wish only to clearly demonstrate that there is already a rich tradition of thinking critically and historically about monogamy *qua* social system.

However, these authors, and these writings, are far from the first to reflect on monogamy and its concomitant pathologizing of excess – of desire, sex, and partners, which has a long-standing tradition ( von Krafft-Ebing, 2011) and plenty of unfortunately contemporary supporters ( Cardoso, 2014; Grunt-Mejer & Łyś, 2019). This pathologization is, evidently, deeply gendered along the lines of the patriarchal sexual double standard, and deeply racialized along the lines of White purity ( Fuentes, 2012; Patterson, 2018).

And yet – we can track how contemporary approaches by those who oppose or reject monogamy went through a series of naming processes that, by and large, do not directly connect to the historical ballast of those pathology-adjacent terms. For example, the ‘homosexual’ was, as a term, floating between being a medical term and a reclaimed term, before it fell out of acceptable use. The ‘swinger’ was not named from a pathology; and the ‘polyamorous’ was most definitely also not ( Cardoso, 2011), nor was the ‘relationship anarchist’ ( De las Heras Gómez, 2019). Schudson and van Anders (2019, p. 362) make the same point about the emergence of terms like ‘demiromantic’, while acknowledging that such a term “borrows the grammar that affords particular labels scientific authority”.

This is not to say that these terms cannot be problematized. As I mentioned before ( Cardoso, 2010), one of the most famous contexts in which the word “polyamory” was coined (the Usenet mailing list created in 1992) was arguably sex-negative, in that Jennifer Wesp made a point to mark that the intention was to *not* discuss issues related to sex. It is not for naught that it was called poly *amory* – after all, love is more acceptable than sex, since it is further away from, as noted above, pathology-adjacent terms like ‘hysteria’ (and its emphasis on disciplining women’s bodies and pleasures) and ‘hypersexuality’, both focusing on notions of excess.

The emphasis on love is not politically neutral or accidental ( Klesse, 2011); it creates very clear narratives about a proper polyamorous identity ( Cascais & Cardoso, 2012), and needs to be read as a response to stigma, as well as a reinforcement of amatonormativity (i.e., the idea that romantic love is a universal, and universally desirable, human experience) ( Olsson, 2022; Scherrer, 2010) and a cornerstone of polynormativity (i.e., the idea that there are superior and inferior ways of living non-monogamously, and that polyamory constitutes a general best practice) ( Pascar, 2018; Zanin, 2013). These novel identities (e.g., relationship anarchist, polyamorous) are still directly related to, and feeding into, culturally and historically hegemonic discourses around gender, sexualities, and relationships overall (namely, sexualization, individuation, and psychologization ( Cardoso, 2017)).

The importance of self-naming, as well as the potentials and limitations of (existing) linguistic resources, was one of the first things to catch academics’ attention in the early days of studying consensual non-monogamies withing social sciences ( Ritchie & Barker, 2006) – as well as one of the reasons why I, coming from Communication Sciences, chose to research communicative processes as forms of producing polyamorous identities. I speak, not against taxonomies, but in favor of their critical deployment.

Because research often needs to talk about broad tendencies that can be heavily influenced, but not totally dictated, by specific cases or instances of a given manifestation of that selfsame tendency, research needs umbrella terms – hypernyms, to be more precise – that can have a more clearly defined taxonomical nature or, contrariwise, a looser overarching connective element that does not neatly fit into a precise taxonomically hierarchical structure.

As I have noted above, hypernyms are twice enmeshed with the issues raised above regarding the politics of naming – they produce (the potential for) identity, but they also order and organize existing identities, often from a top-down, privilege-based, speaking position. This is not to say that such privilege is absolute or that members of those groups do not partake in these academic processes, of course, but rather that they/we never do so from a position of pure subordinate-group-belongingness, and that they/we need to constantly renegotiate the impacts and implications of how they/we occupy multiple and often ambivalent positionings ( Cardoso, 2021), (e.g., activism and academia).

We see that the hypernymy activity around contemporary forms of relating has struggled with the same issues that Jennifer Wesp self-consciously posted about when proposing the creation of alt.polyamory – she didn’t want to use a hyphenated negation ( Wesp, 1992) to describe something positive, and so advanced “poly-amory” as a proposal. However, about 18 years later, the (academic) hypernym created had frustrated Wesp’s plans: the first scholarly edited volume on the topic was “Understanding *Non-Monogamies*” [my emphasis] ( Barker & Langdridge, 2010); in 2015 the “ *Non-Monogamies* and Contemporary Intimacies Conference” was created [my emphasis]; most academic jargon coalesced around “CNM” – Consensual *Non-Monogamies* [my emphasis].

This is understandable – on the one hand, researchers soon realized that “polyamory” was a portmanteau (a hypernym) of what ended up being various relationship practices and configurations, but also an identity that people and communities were taking up (as mentioned above); on the other hand, they also realized that it was insufficiently encompassing of other forms of relating that were also in tension with mononormativity (e.g., swinging ( Gould, 1999; Matsick *et al*., 2014; McDonald, 2010)) and how society at large was in fact able to perceive those differences. The topical thing connecting those varied practices, identities, lifestyles, and so on, was this refusal of the strictures of mononormativity – hence, non-monogamy or non-monogamies.

The other part of the umbrella term most used in academia, however, is equally important: *consensual*. Just like poly *amory*’s name cannot be fully understood without attending to stigma, so are we unable to understand the *consensual* part of CNM without attending to stigma. As research has shown repeatedly, stigma around CNM revolves around its approximation to what is usually called ‘cheating’ – that is, extradyadic sexual/intimate/romantic involvements in the context of monogamous relationships that do not contemplate these involvements as valid or ethical behaviors ( Balzarini *et al*., 2018).

Taking this into account, it is easy to see how polyamorous (as well as other forms of non-monogamous behavior that do not exist in the context of ‘cheating’) identity discursively frames itself around consent, similarly to how kink/BDSM-identified people distance themselves from violence and abuse through consent-based narratives ( Cascais & Cardoso, 2012; Gusmano, 2019; Ling *et al*., 2022; Mogilski *et al*., 2019; Portwood-Stacer, 2013). We see this focus on ethical aspects come to the fore when we look for people’s lived experience and perceptions of how they define their relationships, experiences and identities, sometimes in positive, sometimes in negative ways ( Cardoso *et al*., 2021; Pascoal *et al*., 2014; Séguin, 2017).

As the consensual aspect of these relationship practices and identities was so central to differentiating them from existing normative systems, ‘consensual’ became the analytical gesture of boundary-keeping used by researchers to demarcate their work from work on “cheating” or “infidelity” – even though there are potential overlaps. However, it also resulted in potentially reinforced and exacerbated stigma against ethnic and religious minorities ( Rambukkana, 2015a; Rambukkana, 2015b; Vasallo, 2015).

The story, though, does not stop here. As the CNM label kept moving between activism, academia, and people in various relationship configurations, and as the cultural conversation around consent continually evolves, the ‘consensual’ dimension of the CNM label has been questioned. This has been happening in at least two different ways – both stemming from the same basic premise.

Precisely because the terms poly *amory* responded to stigma, and *consensual* non-monogamies did as well, they are infused with a positive moral connotation, while pertaining to be mere descriptors (and thus morally neutral). There have been two proposed solutions to address this.

The first proposed solution: as I, and others, noted elsewhere ( Cardoso & Klesse, 2022), this has given rise, first, to a series of proposed alternatives, such as *ethical* non-monogamies (ENM), or *responsible* non-monogamies; but also to alternative naming systems that do not really serve the same function or purpose of a hypernym and/or are not clearly defined in a way that is analytically useful, like designer relationships ( Michaels & Johnson, 2015) or novogamy ( Ferrer, 2021).

The second solution: to *remove* the qualifier – any qualifier – from the hypernym. Monogamy is not commonly prefaced by “consensual” in the literature, or in public discourse so, the argument goes, why would we need to preface non-monogamy like so? Such a prefacing seems to validate and reassert the stigma against various forms of non-monogamy ( Travers, 2021).

**Figure 1.**
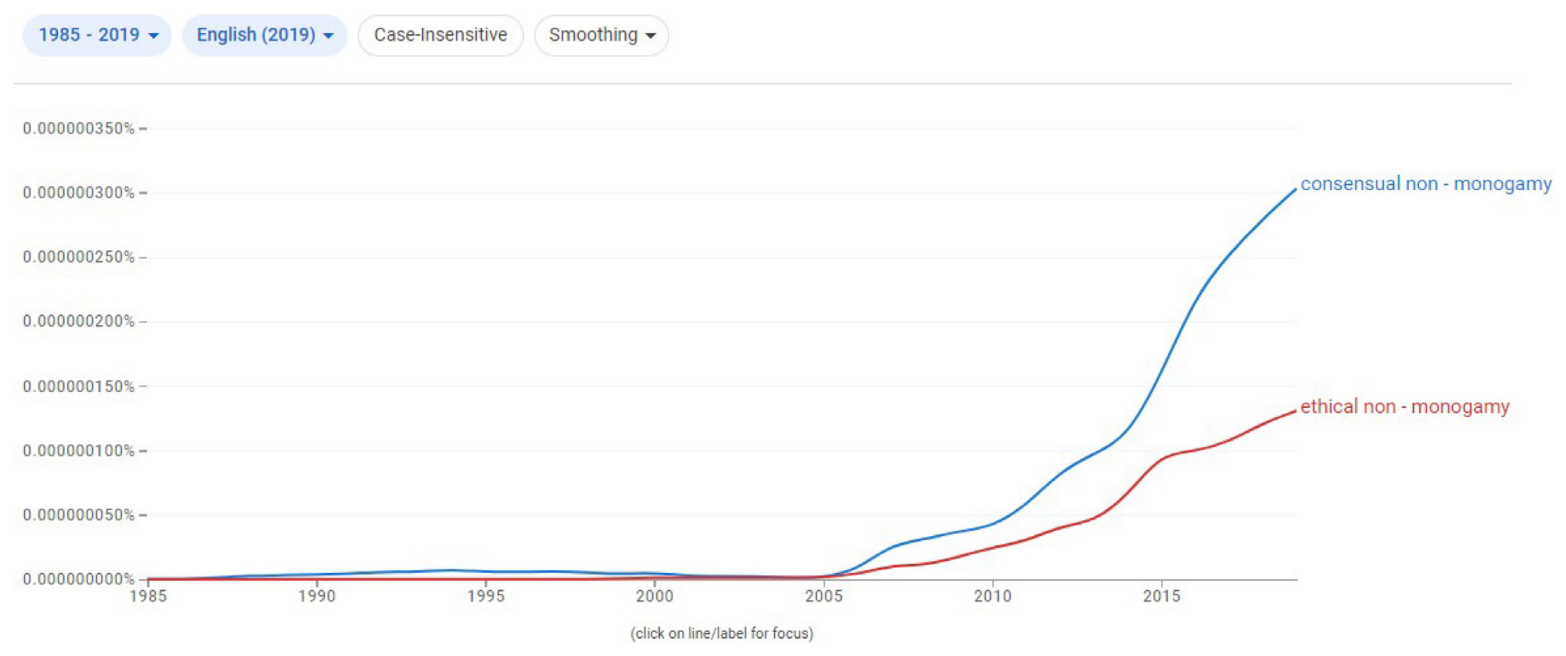
Google N-gram comparing the evolution of "consensual non-monogamy" versus "ethical non-monogamy". Generated March 8th, 2024, by the Author.

**Figure 2.**
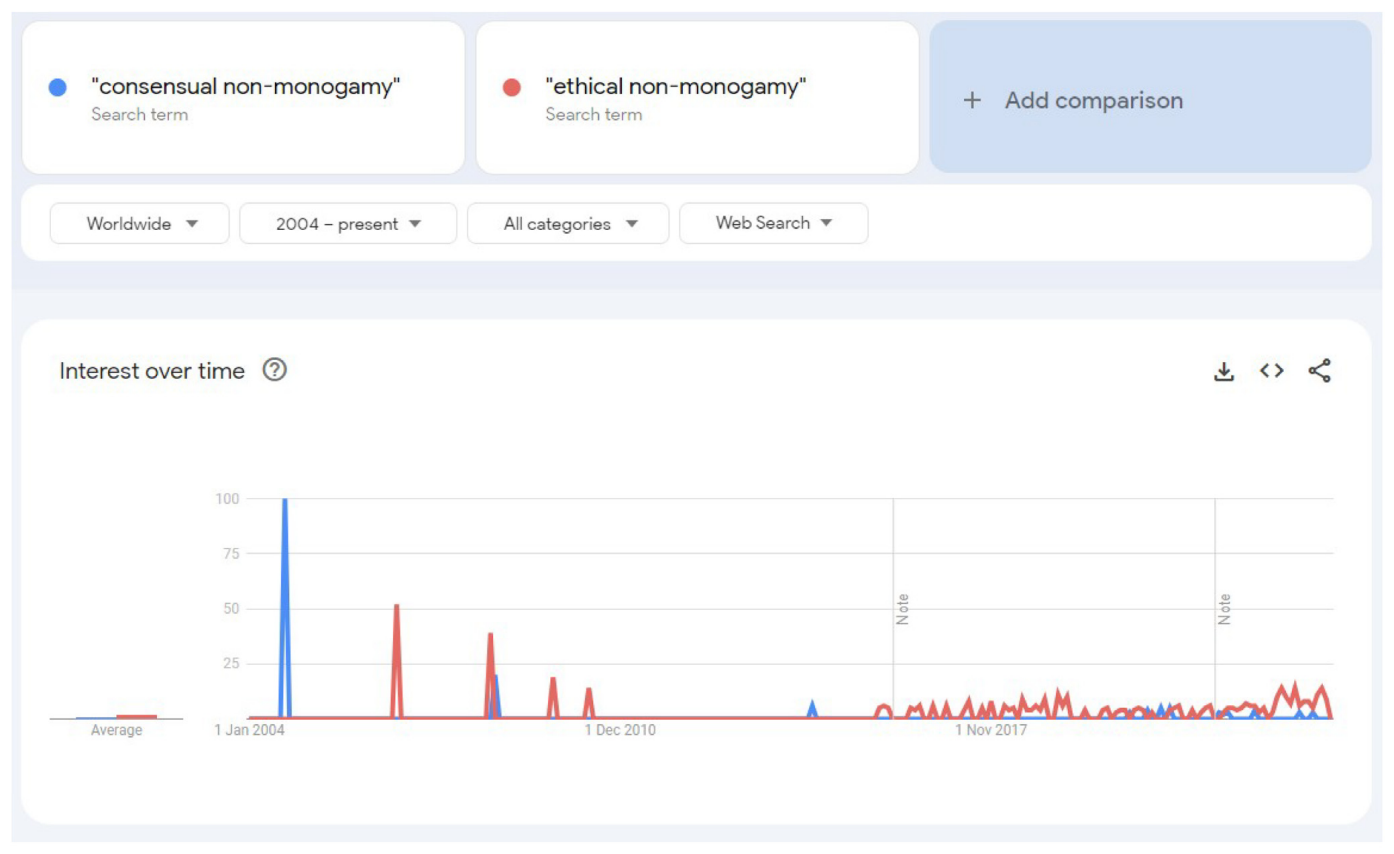
Google Trends results comparing "consensual non-monogamy" versus "ethical non-monogamy". Generated March 8th, 2024, by the Author.

This discussion is ongoing, especially within certain subsets of the community and within certain linguistic contexts – though it has taken a (dangerously) interesting turn, by way of how forgetting works in contemporary social movements. That is to say, a very recent blog post about the topic ( Brooks, 2024) talks “about replacing the ‘ethical’ in the phrase ‘Ethical Non-Monogamy’ with the term ‘consensual’”. I call this dangerously interesting because it inverts the direction of the trend and the historical record available, as it can be seen below in Figure 1 and Figure 2. Figure 1 shows very early traces of CNM in the 1990s without any such equivalent for ENM, as well as a faster take-off from the mid-2000s; and Figure 2 makes that more evident by showing a CNM spike also in the beginning of the mid-2000s. The shift away from CNM and into ENM has become so intense within these communities, that it has generated the notion that ENM arose first and CNM was created in response to it, rather than the (empirically verifiable) opposite.

Even if we disregard whether CNM or ENM came first, or has become more popular, the discussion is still relevant, because the replacement suggestion ends up solving none of the issues that I have raised here: ‘ethical’ and ‘consensual’ are both imbued with morality. As I will later address, this type of terminology joins together facts and values, while seeming to be, at the same time, purely descriptive ( Williams, 2008).

Moreso, as another relevant blog post notes ( Turner, 2019), one of the reasons why CNM should, in the author’s opinion, be used, is because *academia* uses it – generating a self-fulfilling prophecy of academia impacting activist and laypeople’s language, rather than just describing or empirically “reflecting” it.

Similarly, academic work behind naming conventions like “designer relationships” or “novogamy” can have deleterious effects on communities, by constraining terminology, by impacting terminology that might be more readily used in policy-making (in a way that is contrary to what communities wish or strive for), and many other examples. This is not to say that communities are homogenous, speak as one, or even have a centralized system for terminology-choosing, nor is it to say that academic use of certain terms in inherently good or bad. I wish only to signpost here the complex ways in which the respectability of science and academia plays a role in how its use of certain terms can have far-reaching consequences, both positive and negative. I will return to this issue below.

It is beyond the scope of this paper to evaluate the merits of the arguments proposed for using CNM versus using ENM, or even to fully account for the nuance of that (relevant) debate, just like it is beyond the scope here to fully describe or offer a more cogent and constructive critique of other terminologies. I wish only to note that: 1) such a debate exists and is central to these communities, 2) it does not fully address the needs that academics and scholars have when it comes to using hypernyms.

The second proposal – disavowing any qualifier to be used before non-monogamy – is different, however, in that it engages directly with the same issues I address above: namely, the origins and effects (conceptual and practical) of using morally-charged words for supposedly descriptive terms. As Claire Louise Travers (2021) notes, this subtly validates the stigma against non-monogamies (because it is done in response to it), places a moral burden of perfection on those who engage in non-monogamies not predicated on dissemblance, and it allows for the assumption that anything done under the aegis of ethical/consensual is, by definition and *a priori*, ethical and/or consensual and thus undermining the need for continued engagement and reflexivity about moral positionings.

While the removal of any kind of qualifier would address these points (especially considering that, again, qualifiers are almost never used to talk about monogamy), it also does not solve the *academic* problem of when to use certain hypernyms. It is not that researchers cannot use “non-monogamy/ies” as a hypernym, but rather that researchers *already* use it as a hypernym, whose corresponding signified (in the de Saussure (1959) sense, meaning the actual real-world thing that is meant by the word) is wholly different from what is meant by when “CNM” or “ENM” is used, both in academia and without, since the former includes non-consensual relationship structures and the latter (supposedly) does not. Here, CNM/ENM is a sub-set of non-monogamies. A study, or an activist group, that wishes to focus on only some forms of non-monogamies but not others, and that does not have a pre-defined set of identities or specific practices that it wishes to encompass, but does have specific identities or practices that it wishes to *exclude*, will have an even harder time by exclusively using “non-monogamy/ies” as an umbrella term.

To be sure, these discussions are never *only* about terminology, but about how terminology is integral to the constitution of certain subjectivities, how they are part of the process of *assujetissement* mentioned above, and involve power.

## Reflecting on academic acts of terminology-creation

As mentioned above, coining words is a powerful action, just as denying someone, or some group, the right to do so, is no less powerful. Academia is centrally preoccupied with terminology, and thus the reasons, the processes, and the impacts (intended and effective) of which terminology gets created must be equally central to the coining of terminology.

There are many reasons why new terms get created, and many effects those terms can have. To be clear: there is nothing inherently different about new words, as compared to already-existing ones, and it was a paper on new words ( Ritchie & Barker, 2006) that partially set me on my own academic path. However, coining new or changed terminology is an enactment of power that carries with it the potential, promise or illusion, of *changing things*, and so a series of questions must be asked – who is this new or changed terminology serving? What is its main function at the time of creation, and who is centered by that function? How does new terminology interact with existing power structures (inside and outside of academia), and how easily weaponizable is it against the communities it addresses or is about?

To do this, we must pay special attention to the specific systemic nuances involved – on the one hand, hegemonic frameworks and ideologies that make certain worldviews seem more self-evident, while others are subjected to epistemic violence and injustice ( Fricker, 2007); on the other hand, the system of rewards and punishments that surrounds contemporary neoliberal academic work, and how it fosters a system based on visibility, uniqueness and high-speed circulation.

Starting with the latter, and as others have noted, the neoliberal turn in research and academia has palpable effects on research processes and publishing practices. Being published is important – hence the infamous “publish or perish” saying. However, being *cited* is increasingly fundamental and more valued when it comes to accumulating status and negotiating one’s position within academia ( Yin & Mu, 2023). This is evident in concerns surrounding journal indexing, journal ranking and impact factors, h-indexes, and much more (all of which are direct indicators of citations used as proximal indicators of quality or relevance). In fact, the specific impact of being *cited* as part of a growing trend of accumulating social status within academia is possibly the driving reason behind the phenomenon of “sneaked references” ( Cabanac & Besançon, 2024), which is when authors from one academic article insert invisible citations in the document to bolster the apparent visibility of those cited invisibly (who might be themselves, or others); as Cabanac and Besançon (2024) aptly state, “[c]itation counts heavily influence research funding, academic promotions and institutional rankings”.

The pressure to be cited then turns into potential considerations about the marketability or visibility of what we publish, as it might impact one’s professional prospects, job security and stability, career progression, and more. Even without resorting to academic malpractice or misconduct, creating catchy titles, carefully curating your keywords and the contents of your abstract, and minding impact factors when deciding where to publish are examples of this, and they all play a role in how cited academics are.

Creating highly citable, catchy, easily-applicable new *terms* or *concepts* is (or can be) another immensely effective strategy – when it works, and when one is epistemically, socially and historically situated in a way that facilitates this dynamic (for counter-examples, think only on how many concepts coined by racialized women scholars got reattributed to more privileged academics, amid a culture of academic extractivism ( Jekanowski *et al*., 2022), but also how certain seemingly academic narratives gain traction *because* they are so easy to viralize, in spite of their lack of rigor ( Lieberman & Schatzberg, 2018), as was the case with Maines’ *Technology of Orgasm* ( Meyer & Fetters, 2018)).

While the odds of this system are clearly stacked against minoritized groups (even within academia) and their epistemic claims, it does not mean that such strategies cannot be deployed to pursue terminological changes that have restorative impacts for marginalized subjects, that follow the interests and rights of disenfranchised subjects, and that allow for new / different / better / more inclusive intellectual and academic work to be performed. In fact, many terms that have gained wider circulation in recent years (e.g., ‘gender performativity’, ‘intersectionality’) have benefited from this dynamic. If communities (more often than not, nowadays, organized around digital spaces and platforms) create and make visible new terms and identities “to articulate subjectivities that are harmed by [hegemonic] systems and spark creative forms of political and relational thinking” ( Schudson & Van Anders, 2019, p. 365), then it is possible for academic processes to bolster that subjectivity articulation.

This requires, however, that we return to the first point raised above, regarding hegemonic frameworks of producing science. As Sari van Anders *et al.* (2023) note, based on previous work done in the field of feminist and queer epistemology of science ( Haraway, 1988; Harding, 2006; Harding, 2015), feminist and queer scholars have developed a series of profound critiques to the *praxis* of science, and its gendered-ness – a call to take into account who does science, what counts as science, but also what is *supposed* to be the role(s) of science as a socially embedded process. In ways that drink from, and are orthogonal, but not wholly dissimilar, to critiques of the relation between technology and science ( Heidegger, 1995) and, as mentioned above, power-knowledge ( Foucault, 1994b), feminist and queer ways of engaging with science are predicated on the idea that scientific processes do not merely *describe* the world, they *inscribe* it, they *construe* it. This is why, for instance, Ana Cristina Santos (2012, p. 245) brings to the fore the need for queer public sociology – “ a shift in the research ethics underpinning epistemological and methodological choices to the extent that contributing to social justice becomes a central aim of knowledge production […], particularly relevant when the topic of research has historically been subject to discrimination and inequality”.

Under the aegis of such considerations, and focusing on the aspects of queer science that van Anders *et al*. (2023) highlight – construction, openness, challenge, and multiplicities – we can look at the specific act of creating new terms, new classification systems, or new hypernyms, as an especially salient situation where the (in)existence of such characteristics needs ascertaining. As we create new terms, especially new hypernyms, a thorough analysis of power dynamics (and foreseeable effects and consequences) is mandatory, not only on the grounds of morality ( Burgess & Plunkett, 2013), but as another layer in the scientific process, necessary to ensure that our work “leads to a more empirical, accurate, and just knowledge, including of gender/sex/uality, and can shape science and its impact on the worlds we live in” ( van Anders *et al*., 2023, p. 15). Sari van Anders’ work is paradigmatic in this regard – her “Sexual Configurations Theory” ( van Anders, 2015) could very well fall into this trap of high citability, but the original paper is thorough in its clear articulation of *both* continuities and discontinuities with existing terminology (rather than focusing only on the discontinuities, the differences, the changes), in its interoperability with other terminological systems, in its scope of usage, in how minoritized communities might engage with it; and subsequent work done with other colleagues has focused, for several years now, in ascertaining just how that term is being taken up, and on how, why and for whom it can or cannot be useful, rather than assuming that it will be useful just because it can be useful ( Abed *et al*., 2019; Beischel *et al*., 2021). Here, we are in the realm of “conceptual ethics” ( Burgess & Plunkett, 2013) – that is, considerations that can help us think about how the concepts we use impact *how* we think, which, in turn, impacts the world; and that can help us make more evident when a disagreement in the use of a concept has to do with ontological issues or with the usage of certain linguistic resources in certain contexts.

To clarify – it is not how catchy, or citable, a term or a hypernym is, that is a problem unto itself. The issue relates to the relative positioning of those coining the term, those benefiting from the term’s diffusion, and the underlying reasons that motivate and serve as justificatory of the term’s coming-into-being and its deployment. Relevant (new) terminology that materially and socially pushes back against existing inequalities and is catchy is not the issue at hand here – that is one mode of mobilizing power in resistive ways within academia. However, when catchiness or citability become *goals in themselves*, at the expense of, or in disregard of, the communities that they are supposed to address, and when the measurable material benefits mainly flow to the academic *qua* academic, sometimes even imposing themselves on those same communities in a way that forecloses or erases existing marginalized narratives, concepts and ideas – *then*, we have a mode of power-knowledge deployment that is in violation of the ethical obligations placed upon academics, and in service of neoliberal forms of symbolic violence.

Part of this reflexive exercise comes from my own experience – I have also attempted to create catchy terms. At the time, I did think that it would make it easier to point out a specific element in what I was addressing (in this case, the connection between relationships and ‘the political’, and how to think politically about relationships ( Cardoso, 2015)). Looking back, though, I see very little difference that the new term could make or have made, and little – if any – change to the existing body of knowledge at the time. More recently, I have had the pleasure and privilege of working on ways to better understand how people make sense of their experiences, or how they define things, and I have been lucky and honored to work alongside, and under, others who have similar priorities. My and our preference has been placed in understanding the usages of existing terms ( Cardoso *et al*., 2021; Cardoso *et al*., 2023; Tselenti *et al*., 2024) – and the consequences of those usages – to comprehend what other intellectual work can be done or what critiques ( Cardoso, 2022) we can derive from that and incorporate into academic practice.

Considering this lengthy critique about creating and managing terminology, this paper seems almost comedically out of place, since it literally is a proposal for terminological change. I wish to address this head-on, by noting two things: 1) while I have no control over what others do with this proposal, I have no interest (nor do I *see* any usefulness) in having this proposal be applicable outside of academia, as it is a merely technical solution or response to a terminological and analytical problem, hopefully sufficiently grounded to be useful; 2) I have purposefully chosen to publish this in the Open Research Europe platform, precisely because the article, as well as the peer reviews, are all publicly available, and because the platform not only allows, but encourages, the author to submit new versions of the article, thus maximizing the transparency of the process and to track the origin of new ideas, improvements, suggestions, corrections, and so on.

A disclaimer, then: I do not think that this terminology should be the basis of a novel understanding of non-monogamies, nor that it should be a new paradigm with which to interpret relationships overall. Instead, my intended contribution is in the order of what some have called “little t theories” ( Schneberger *et al*., 2007). These serve to drive small-scale and limited-reach research, and help connect elements from more encompassing (or “big T”) theories. In this case, I merely wish to engage with a technical-theoretical difficulty: how to use umbrella terms, and which umbrella terms to use, when doing research on relationship types and practices that sit outside the mononormative system. This scope of application is intentionally narrow, as to minimize the ways in which communities of people who engage in varied forms of relationship configurations make sense of their own experiences; however, at the same time, I hope to be able to – both inside and outside of academia – raise awareness of how morally-loaded the terms that are in circulation have been (or become), and the attendant risks for scientific praxis and for everyday relationship dynamics.

## The proposed terminology

Considering the issues of specificity and morality that constitute the fundamental challenge I wish to address here, it follows that morally-charged words are ideally out of the question. However, any behavior can have, in a given social context, an ethical or moral connotation. Therefore, while there are terms which can be seen as only descriptive, very often it is also the case that both facts and values are encoded within a given term ( Williams, 2008, p. 129), regardless of whether we intend for that association to happen or not (and this may be especially so when classifying or naming things within an academic or scientific context).

At the same time, there is ultimately nothing ontologically unique about polyamory, or relationship anarchy, swinging, etc., *qua* forms of relating. Therefore, terminology that can help us organize relationships should be equally applicable to monogamous relationships and, even, help to illuminate or put under the spotlight its current Global North-based hegemonic status.

In this section, then, I will first outline the proposed terminology, provide examples for each of the categories, and further explain how this terminology does not necessarily rescind or replace existing categories or hypernyms, but rather opens an orthogonal categorical system.

### Tripartite categorization around explicitness

Rather than using a binary system (monogamy/non-monogamy; consensual/non-consensual; novogamy/ *archeogamy* ^2^), I use a 3-category system, which has as its nodal point *explicitness*. To clarify what I mean, it is first important to address the meaning of “explicit”, since that is also the basis for my proposal.

According to the online version of Merriam-Webster (2024), the word comes from the Latin “explicāre”, meaning “to free from folds or creases, unroll, disentangle, spread out, set out in words, give an account of” – the prefix “ex” meaning the undoing of, and “plicāre” meaning the act of folding or bending. We see, already here, that communication plays a part in the (metaphorical) meaning associated with explicit (hence, why we also have “explain” or “explicate”).

It is also outside of the scope of this paper – not to mention, an impossible undertaking in such a short document – to present a full account of the myriad theories of (human-to-human) communication, and even more so if we were to include pragmatics on top of it. Many different theories exist, across both Communication Sciences and Pragmatics, to account for how people create meaning for themselves, how and why they attempt to transmit meaning, what roles the recipients of meaning have in the success of a given communicative action, what roles grammar, culture, and different media have in how that happens, and even how we can define what constitutes a successful communicative action. Part of this, in fact, can also cogently be argued to require phenomenology applied to communicative situations. Previously mentioned authors, like Saussure, but also others like Austin, Searle, and many more, have dedicated untold time and resources to delve into these topics (e.g.: Adler *et al*., 2020; Lanigan, 1977). I raise these questions here, not to fully address them, but to: 1) signpost a few underlying assumptions that I will be using, 2) signpost some potential objections that can be raised from the usage of those assumptions, or from departing from other theoretical traditions, 3) move to specify elements that are fundamental to my analysis, and that are only present in some of those same communication and pragmatic theories.

The assumptions:

- that intentional communication is *possible* (i.e., in this context, that it is possible for speaker A to convey meaning to speaker B in such a way that speaker B will perceive this meaning in a way that is functionally or sufficiently similar to what speaker A wished to convey);
- that non-deceitful communication is *possible* (i.e., that the meaning that speaker A intends and attempts to convey is willfully functionally similar to the meaning that speaker A holds to be true);
- that an evaluation of the success of the communicative act can *possibly* be reasonably arrived at by speaker A and by speaker B, and that it is *possible* that both speakers believe they are in agreement about the evaluation of the success of the communicative act (i.e., in this context, that speaker A believes to have correctly been understood by speaker B, and that speaker B feels they understood speaker A);
- that there is no communication without sociocultural and linguistic *context* (i.e., that conveying meaning necessarily engages with the (in)existence of shared communicational codes, cultural references, and so on, which often sit outside the communicative act itself, as well as, primordially, relations of power between the speakers).

Many of these assumptions are (over-)simplified from what is usually known as reflexive communicative intention; communicative intention is usually attributed to Grice, but many authors have expanded on the topic (e.g., Green, 2021), and I follow here the approach taken by Recanati (1986).

To simplify Recanati (1986), if speaker A *asserts* something to speaker B, expressing something that speaker A holds to be true, with the open intention that speaker B comes to hold that thing to be true too, and the thing said gives speaker B reason to believe in that thing that speaker A holds to be true, and speaker B recognizes speaker A’s intention to say something that openly conveys the wish to give speaker B reason to believe in that thing that speaker A holds to be true, and speaker B recognizes that open intention – only *then* do we obtain what is termed reflexive communicative intention. I note that reflexive communicative intention includes other phenomena than assertions, which I do not focus on here.

The exact content of the assertion is not defined by any of these assumptions or by reflexive communicative intention. It is heavily dependent on context, on who the speakers are, on what knowledge they have in common or not, and importantly on the relations of power that obtain between them and how those might impact this process.

While this might seem like a detour from the main topic, reflexive communicative intention is at the heart of how I am mobilizing “explicāre” – to set out in words or give an account of something, but in a way that obeys the constraints of reflexive communicative intention. That is to say, my use of *explicit* in this categorization requires self-reflexive honesty, transparency, and openness about the wanting to be explicit, *but also* (and here, we partially move beyond reflexive communicative intention), a modicum of concern with reasonably acting/speaking in a way that attempts to ensure successful communication, considering the aforementioned circumstances in which that communication is taking place.

A few examples: if speaker A knows, or has good reason to assume, that speaker B does *not* know what “polyamory” means, and says “I am polyamorous” (while not attempting to clarify what that means), this does *not* constitute “explicit” for the purpose of this classification system; if speaker A knows, or has good reason to assume, that speaker B is very knowledgeable about the topic and terminology around CNM/ENM, saying something like “Right now, I am in a primary relationship with two other people” *does* constitute “explicit” for the purpose of this classification system. Likewise, in the first scenario, a clarification or definition of polyamory, for example provided upon request by speaker B, would turn the interaction “explicit”; in the second scenario, the (legitimate) assumption turning out to be false, and speaker B making no efforts to seek clarification or disambiguation when faced with a clearly worded utterance that they could not understand (or mistakenly assuming they had understood the utterance), would *not* make the communicative interaction anything other than “explicit”.

These examples will hopefully clarify not only the practical meaning of explicitness, but also allow for a more nuanced understanding of the shared responsibility of interpersonal communication. Precisely because responsibility for clear communication cannot be unilateral, but is often also not perfectly symmetric, and because perfect communication cannot exist, day-to-day interpersonal communication requires some degree of proactivity and metacommunication on both sides, and unsuccessful communicative acts cannot *a priori* be imputed to only one person.

Therefore, explicit communication can exist even if it is unsuccessful, and seemingly (or, rather, semantically) ‘explicit’ communication can, in fact, turn out to be, by action or inaction, *not* explicit, because of various contextual reasons. However, considering that my proposal is for a tripartite system, arranging different scenarios into explicit or not, is insufficient. What follows is then the condensation of my proposal.

### Classifying explicitness: Explicit, Implicit, Covert

In order to prevent oversimplification of interpersonal communication, and to account for power and resource differentials, as well as the differing responsibilities that a speaker and a listener have, while still making the classification system *useable and practical* in a research scenario (be it data collection, analysis, or reporting), I propose the following organizational structure (reliant on the notion of explicit explored above):

- **Explicit** – relationships, or relationship structures and networks, or relationship paradigms, or relationship discourses, or assertions of identity or behavior, which are predicated and based on outwardly reasonably unambiguous communication of the fundamental (as applicable) paradigms, rules, expectations, structures, networks, practices, discourses, or similar, between all the involved parties;
- **Implicit** – relationships, or relationship structures and networks, or relationship paradigms, or relationship discourses, or assertions of identity or behavior, which are predicated and based on a willful and/or careless (by action or inaction) lack or absence of fully explicit communication of the fundamental (as applicable) paradigms, rules, expectations, structures, networks, practices, discourses, or similar, between any of the involved parties;
- **Covert** – relationships, or relationship structures and networks, or relationship paradigms, or relationship discourses, or assertions of identity or behavior, which are predicated and based on purposefully misleading and/or misrepresenting communication of the fundamental (as applicable) paradigms, rules, expectations, structures, networks, practices, discourses, or similar, from any of the involved parties to any other involved party, and/or a change in behavior from any of the involved parties that falsifies previously explicit or implicit communication, when it is not accompanied by further communication about that change to all other involved parties.

To again exemplify with the same not-explicit scenarios as before: the person who presents as polyamorous but has no reason to believe they were truly understood by their potential partner would fall under the category of “implicit non-monogamy” if they were to enter in a relationship with that potential partner. Imagining that this person has other partners with whom this was not the case, the entire relationship structure that involved that person would be in a situation of either implicit non-monogamy (e.g., if the rest of the network existed in a Don’t Ask, Don’t Tell (DADT) system and had no rules or assumptions about any metamour dynamics) or covert non-monogamy (e.g., if the rest of the network had a reasonable assumption that everyone involved would only engage in explicit non-monogamy with other potential partners).

This does not mean that everyone would *know* about, be *responsible* for, or be *doing* implicit or covert non-monogamy, but that the classification of the network as a whole must always default to non-explicit (i.e., to implicit or covert, as applicable) whenever there is an implicit or covert involvement. Likewise, this system can be used to analyze subsets of a given relationship network (e.g., assuming DADT, the subset of this person and/or any of their preexisting partners are still in an explicit non-monogamy arrangement), or characterize dyadic connections separately (implicit non-monogamy between A and B; explicit non-monogamy between A and C, assuming DADT). For example, a sister-wife ( Rosengren-Hovee, 2021) that marries into a polygamous family is practicing explicit non-monogamy, and the whole structure/family is equally categorized.

This classification system is equally applicable to hegemonically-situated experiences. Two people who started dating, and did *not* discuss whether that meant sexual/intimate/romantic exclusivity, have a relationship that is categorized as implicit monogamy precisely because the lack of communication and the reliance on assumptions (i.e., their hegemonic situatedness led them to the same conclusion, but it relied on, and was fed by, default assumptions which tend to not be communicated). Likewise, two people who decided to ‘go steady’ but relied on assumptions about what that meant, are also categorized as implicit monogamy. Contrariwise, a couple who has undergone a religious ceremony whose vows contain references to exclusivity, or who has gotten a civil marriage in a country that has anti-adultery laws, is classified as explicit monogamy.

Covert non-monogamy is perhaps the most obvious example: it is the paradigmatic ‘cheating’, or ‘infidelity’, but it is also the violation of the rules, agreements or negotiated expectations of involvement in an otherwise consent-based non-monogamous arrangement; seen from the perspective of all involved, it is covert non-monogamy, but different people occupy various positions in relation to its covertness: it is covert for the person doing the covering-up in a different sense that it is for the person being ‘cheated on’, and if the third party knows about the existing relationship, that is yet a different sense. This example makes clear, I hope, how this system classifies connections or relationships from the analysis of who communicates what how, but not *people* themselves. Covert monogamy might seem strange, but it is not impossible: someone who claims to be polyamorous (or to have multiple relationships) but does not actually consider that to be true, or someone who does so with the covert intent of ‘converting’ a non-monogamous person into monogamy.

### Terminology and issues of moral valuation

This classification system does not intend to replace existing terminology – in fact, it is entirely possible to use them at the same time in order to specify different aspects of relating. The primary reason for this is that explicitness is not tied to the same morally-centered ‘variable’ present in “consensual” or “ethical”. As mentioned before, the existence of moral valuations within terms that are presented as merely descriptive is a central issue for this proposal, and the driving point of contention in the criticism of the use of “ethical” or “responsible” or “consensual” non-monogamies. I turn now to this issue, since it is also present in my classification system, albeit – I will argue – in a fundamentally different way.

The use of the word ‘covert’, as well as the definition that I have provided for it, are in some ways morally-slanted, as even I use the expression “purposefully misleading”. Note, however, that the *second* part of the definition does not require purposefully misleading action, but inaction that renders previous communicated statements false. Evidently, the refusal to update that communication is voluntary and intentional, but the intent to deceive is not necessarily found at the origin of the (initially explicit or implicit) communication or at the act(s) that make it false. It does become morally-connotated after the purposeful misleading element comes into play.

There is a term to address this specific issue – in this instance, “covert” can be considered a “thick concept” ( Williams, 2008), which “express[es] a union of fact and value […] applied [in ways] determined by what the world is like […] and yet, at the same time, [its] application usually involves a certain valuation of the situation, of persons or actions” ( Williams, 2008, p. 129). In thick concepts, then, facts and values are completely connected and cannot be fully separated from one another – there might not be (and often, there is not) a purely descriptive word that can encapsulate what exactly is meant by the use of certain thick concepts. Bianca Cepollaro (2020), in her analysis of slurs (which are, according to her, a specific case of thick concepts), calls these “hybrid evaluatives” (HEs): terms that “carry descriptive content at the level of truth conditions, but they presuppose an evaluative content, i.e., they trigger an evaluative presupposition” ( Cepollaro, 2020, p. 1).

It could be argued that “covert” works here in the same way that “ethical” or “consensual” works in the other classificatory system. However, in those cases, “ethical” or “consensual” are not thick concepts, I argue, but *thin* concepts. Cepollaro notes that the distinction between thick and thin concepts is not fully settled, but offers various possible ways to posit a distinction, one of which is applicable here: “the evaluative content is at-issue for thin terms and *presupposed* – hence, not-at-issue – for HEs, whose descriptive content is instead what is at issue” ( Cepollaro, 2020, p. 7). What this means, in this case, is that “ethical” is the at-issue element of something like ENM, because the distinction is made between the ethical and the unethical, it is its purported morally-acceptable status *as* ethical that makes ENM be ENM; however, in the case of “covert”, that does not apply, since the descriptive element (what was communicated or not, and how) is the at-issue element, and its moral valence (whether being covert is a good or a bad thing, morally speaking, or perceived as such by whoever is using the concept) is presupposed, and can even be hypothetically different in another context without it interfering with the way the classification system operates.

While, then, there is a moral valuation present in the term “covert”, my argument is that the classificatory center of this system is not rooted in the moral aspect itself, but in what is obtained between the utterances and the actions of the people involved, within a certain context and under certain limitations and constraints. Thus, the issue is, properly: explicitness.

I hope that the terms used can also metaphorically help clarify this: if “explicit” means taking out the imperfections and folds (in a communicative sense, to make things plain [i.e., *flat*] and visible), then “implicit” means failing to take out (or even adding) those imperfections and folds that obstruct things from (communicative) view. “Covert” can be understood by the very same metaphor: to cover certain elements of potential communication, or of actions, so completely that they become invisible, totally enfolded; or to fail to remove those folds and imperfections that might make the communicative view unobstructed. If “implicit” means that some things get hidden or unseen in the folds, “covert” means that only the folds are visible, but never what is underneath them, and that those things are completely out of sight and cognizance (or, at least, are meant to remain so, by one of the parties involved at least).

Furthermore, and even though I just called them *thin* concepts, both “ethical” and “consensual” have a plethora of definitions – and the way laypeople understand and conceive of how these things are to be done in actuality is also equally complex (see, for instance, the differing ways in which moral standings and actions were seen as part of people’s definition of polyamory ( Cardoso *et al*., 2021)). While it makes sense to seek out better formal definitions, a classification system that pays heed to how people perceive their experiences must hold space for those very same perceptions, and so it is important to separate – in order to better understand – what role explicitness plays into peoples’ ideas of “consensual” or “ethical”.

### Using the explicitness classificatory system

What follows is a series of examples of how this proposed classification could co-exist with other attributes, by making use of multiple (internally inconsistent) perceptions of what “ethical” and “consensual” might mean. It also intends to show how certain situations, which might be difficult to characterize or classify using the other existing terminologies, are made easier to classify using this explicitness system. I will resort to situational stereotypes in the hopes of more easily capturing what the main similarities and differences are.

Example 1: Two people meet in a bar, get sexually involved, exchange contacts and occasionally see each other again and have sexual involvement again; they always include some form of safer sex practice, and never really discuss their connection in any depth. This could be seen as ‘cruising’ or ‘dating around’ (or the more judgment-laden ‘sleeping around’). Within this classification scheme, this is an evident case of implicit non-monogamy: they never really discuss anything, and they both draw on various assumptions and unspoken rules based on a shared cultural background. Within the CNM/ENM framework, this would be harder to define: could we consider this a relationship if they don’t address it as such?; is this serial monogamy (just because there’s no orgies, maybe)?; is this a form of non-monogamy, and if so, is it consensual if they never speak about it just because it so happens that their assumptions are correct?.

Example 2: A couple constituted by a cisgender man and a cisgender woman puts a personal advert online looking for “a third”, in what is sometimes referred to as “unicorn hunting” ( Johnston, 2022), and which many define as being inherently unethical (whereas others consider it ethically unproblematic, as long as there is a priori knowledge of that exact dynamic), and a cisgender woman responds and engages with them. Here, we have two routes with the terminology I have proposed: either the way the advert is written is very clear about the roles and ‘rights’ of all those in the relationship, at least to the respondent (and thus, explicit non-monogamy), or it is not (and thus, implicit non-monogamy). Using existing terminology paints a more muddled picture. For example, if we were to take the stance that “unicorn hunting” is inherently unethical, then we would have implicit (unethical) non-monogamy. But there are interactions of this sort that do not necessarily constitute “unicorn hunting”, even if they involve a heterosexual couple seeking a bisexual woman for a partnership of some sort, or we might not consider “unicorn hunting” to be inherently unethical – in those cases, it would be explicit non-monogamy (if the rules are clear), or implicit non-monogamy (if they are not), but also always ENM/CNM. If we were to use differing notions of consent (e.g., the ability and act of agreement to something), and ethics (e.g., attendant to power imbalances, etc.), we could even have implicit consensual unethical non-monogamy (though I am aware that this is probably highly unlikely). To clarify, I am not espousing any specific attitude or valuation of “unicorn hunting” or any situation that might be perceived as such – only noting the ways in which different classificatory systems allow us to arrive at different aspects of what we are classifying.

Example 3: Two people start dating and there is even a very lovely and touching candle-lit dinner where one of them asks the other to be their partner. They clarify their status, they talk about their wishes and desires for the future, but they are both reliant on a mononormative script about what it is to be someone’s “partner”, and so they both assume that the other one thinks this means a monogamous relationship, and they both start acting accordingly. This would be a case of implicit monogamy under this classification scheme. It could alternatively (using the CNM/ENM framework) be argued to be non-consensual monogamy (as there was no discussion of that aspect in specific), even taken to the point where it could be seen as non-morally permissible ( Chalmers, 2019; Chalmers, 2022).

There is another advantage to the proposed terminology; I would like to argue that it makes it harder to cogently replicate certain analytical separations that reify existing ethnic and racial discriminations. Under this classification system, a Mormon polygamous family, or a non-Western traditionally (but non-legally) polygamous family, are in the same grouping as a fully negotiated kitchen-table style polyamorous constellation – explicit non-monogamy. This *does not* mean that these two things are equivalent – sociologically, psychologically, culturally, or historically –, nor does this classification scheme aim to address and name those differences (quite the opposite, in a way, considering how the (over-)emphasis on those differences has been weaponized against religious and ethnic minorities ( Rambukkana, 2015a)). Yet existing terminologies do not usually mean to include these relationship structures under consensual or ethical non-monogamy, even as they might arguably be so; as such, those terminologies fail at a number of common, real-world instances, or have consequences that, while not intended, reinforce systemic inequalities.

### Applicability

While having a classification system might help with writing papers and book chapters, or running analyses, it is also important to consider data collection, as it turns into one form of academic representativeness for subordinated groups.

I believe that this classification system can easily be incorporated into existing or new surveys. Imagine, for example, a series of Likert scale statements where the respondent is asked to rate their agreement to those sentences: among those, one could read “I have had one or more explicit conversations about sexual and emotional exclusivity”, another could read “There is an unspoken agreement between my partner(s) and I that we are not sexually/romantically exclusive”, yet another could be “My partner(s) believe(s) I am unaware of their sexual/intimate involvement with other people”.

In simpler, yes-or-no, questions, we could also ask: “Have you ever discussed issues of sexual exclusivity with [partner X]?”; “Have you ever attempted to hide information about other concurrent relationships from [partner X]?”; “Do you believe that your partner [X] has the same views on what the right thing to do is, when you meet a potential new partner?”.

These are just indicative and by no means the perfect version of such questions. Rather than provide a definitive guide to its use, I wish only to signpost that there are a plethora of ways in which explicitness can be mobilized from a methodological perspective for questionnaire items. This also applies to other forms of research – semi-structured interviews, for instance, could help flesh out different ways in which people understand and use explicitness, and/or what communicative practices, presuppositions and doubts they have as to what constitutes explicitness.

Overall, there are various research questions that the new terminological framework opens or complements: how do people conceptualize explicitness?; what do they need and what do they do to feel things are explicit?; when and why do they engage in implicit modes of relating?; are there psychological or relationship correlates of different modes of explicitness?; what are the roles that covert monogamy has in seemingly non-monogamous relationships?; how can explicitness be used to understand informed consent practices?

Again, this has no pretension to being a complete or exhaustive list of research questions – the opposite is true, in fact. The goal of this list – and of this paper in general – is to open up the idea so that the scientific community can engage with it and, more importantly, improve it. There are very many resources and studies done on relationship practices, consent negotiation, information sharing within CNM contexts, mononormative stigma, and much more, that can and should be used to improve on this proposal.

### Potential problems

No proposal is perfect, especially in its first iteration. Below, I will address the objections I have so far been able to foresee.

The moral problem: As noted above, it is true that no classification can be ensured to be value-free. However, the main focus on how the classification would be operational is through communicative practices and dynamics, first and foremost. In this sense, the assumptions and the underlying moral connotations that might be attributed to this language are not brought in by its intended use. While it is true that terminology like “covert” is not morally neutral, I hope to have shown that this is partially unavoidable, and that it does not detract from the main advantage of my proposal, which is to prevent the aprioristic determination that some relationships are inherently (un)ethical by means of their naming alone.

The definitional problem: As I hope to have made clear, I do not think that explicitness is wholly ‘objective’ (i.e., observer-independent). Evidently, what some might consider explicit, others might consider to be implicit or even covert. This, of course, can be turned into its own research question. However, that does not resolve the underlying issue, which is that some degree of observer-independence is needed to be able to classify something as being explicit or not. Still, the detail I have provided to the concept of explicitness can, hopefully, make an external observer’s definition of explicitness more operational and empirical than “consensual” or “ethical” – and, as mentioned before, this classification system is made not to be used in direct everyday contexts.

The subjectivation problem: It is self-evident that, yes, a subjectivity (and thus a normativity) can be produced from this classification. However, and in connection with the previous point, this classification system might be of limited value for everyday use and for people to build their own subjectivities around it. Specifically, the fact that this system joins together identities that are seen as ‘desirable’ and ‘undesirable’, and practices that are ethical and unethical (or consensual and non-consensual), might conceivably make it less mobilizable for such a purpose – identities, when they are self-ascribed, tend to signal *positive* things about the subject to a group of people (even if that group is subordinated and seen as deviant by society at large ( Becker, 1997)), rather than create confusion about the subject’s virtues vis-à-vis other forms of socially intelligible values. The ways in which this proposal interacts with existing terms can also bring to the fore the way certain social and cultural norms are, currently, harder to see. As I have shown above, it enables the linking together of experiences that are often (racially) separated, and the separation of those that tend to be (sex-gender-ly) cojoined. It is my intention that this will allow for the queering of research on relationships and intimacies, and for the critical reflexivity needed to denote political and moral judgments around relationship configurations, systems, styles, origins and dynamics.

The methodological problem: While it might be potentially complex to understand and ascertain the conditions of communication under which a certain relationship, or set of relationships, were obtained, I think that the main reason this is not usually applied to the CNM epithet is because a very minimalist, superficial and/or vague definition of “consensual” or “ethical” is used. Likewise, I use a linear definition of explicitness, but hopefully one that is not vague and that is useful for research, both methodologically and in writing up the results thereof.

I hope the objections raised above, as well as new ones, can be used to improve on this proposal through iterative work from various sources, academic and not.

## But why explicitness?

Regardless of the usefulness of this proposal, and the *how* of explicitness, I would like to finalize with the why behind explicitness – and, through it, to actually bring a point of connection to existing classifications of types of relationships.

In most recent advances in thinking about consent, the procedural management of information differentials, not only of the people directly involved, but also between them and the knowable information that might be relevant to them (regardless, or especially, if it is unavailable to them), has become fundamental; likewise, it is not an excessive generalization to say that Western concerns with ethics are often concerned with protecting personal autonomy (often also termed freedom), be it positively or negatively, within a wider framework of power relationships ( Barker, 2013; Bauer, 2019; Cardoso *et al*., 2023; Fahs, 2014; Foucault, 2000b; Levine, 1991).

Both in terms of positive and negative autonomy (or freedom), and as the saying – attributed to varying sources – goes, “knowledge is power”; Foucault makes a similar point when talking about power-knowledge ( Foucault, 1994b), and the ways in which modes of knowing and modes of doing are co-constituted. The knowing subject cannot truly be accidentally knowing – and thus, the subject must come to terms with what it knows. To put it in terms previously addressed, epistemic injustice or violence can only be perpetrated when there is a subject who claims for itself a knowing ( Fricker, 2007) – all forms of violence require the existence of a victim or target of that violence. To address epistemic injustice is already to affirm that there is some *thing* to know, and some *one* to do that knowing, someone being prevented or denied the validity of even asserting the possibility of that knowing.

This, then, requires that things are knowable and known by someone, somewhere, somehow. Whether we call it knowledge, wisdom, information or data (and I do not see these as synonymous, but yet again I must claim such differences are not central to this paper), all of these require some form of communication, and some dynamics around where such knowledge comes from, goes to, through which means (and media), and serving whose purposes or interests. Shaping flows of communication is exercising power, just as speaking truth to power can be a form of resistance ( Foucault, 1999).

This is evident in how discourse around the *ethics* or the *consensualness* of non-monogamies is so often framed around disclosure and communication ( Barker, 2005; Cascais & Cardoso, 2012; Tiidenberg, 2014); the term “disclosure” is especially morally loaded (especially thick ( Williams, 2008)) and still harkens to the stigma surrounding various forms of non-monogamy, but it also shows how powerfully discussions around autonomy frame the work done on ENM/CNM.

Explicitness is also connected, though indirectly, to concepts of ethics and consent – which is why these hypernym systems can be used in tandem, as demonstrated above – but it also focuses on a different aspect of those selfsame dynamics. Explicitness is, seen through this lens, one facet of how power-knowledge operates, though far too limited in scope to stand on its own or to allow for a full account of those operations, or a full extrapolation of existing power relations from explicitness alone. A great power differential might be reinforced through carefully curated flows of information, but it might also be the case that there is no careful curation due to the lack of fear of what might be done with that information. One reason why this classification does work is because of how it only pays selective attention to this issue of power and communication in explicitness (e.g., by refusing an absolutely objectifiable definition of it); that is also the reason for its purposefully limited applicability, and the need to employ it carefully and alongside other theoretical and methodological approaches.

Explicitness captures, when used in this way, something *smaller* than ethics or consent, and is therefore easier to grapple with, easier to mobilize in research practice, and a hopefully less morally-loaded terminology (and thereby allowing for more *direct* – and even explicit - communication of moral and/or social judgement), and thus less able to be used to perform harm.

## Conclusion

In this paper, I have argued that boundaries and definitions are fundamental across all scholarly domains, and that they are acts that involve power; they are power in action. I have argued that this means that any naming convention – whether new or not – carries with it particular assumptions and impacts, and that this is particularly salient in social sciences and humanities, not because definitions are somehow different here, but because they can very easily and directly impact people’s political rights, autonomy, wellbeing and even their own existence. Ultimately, naming is political ( Alia, 2007).

Furthermore, in the social and human sciences (and in those sciences that touch on life), naming is inextricably linked with subjectivities that are, in turn, linked with normative systems – again, systems of power, control, dominance, and resistance; the push between the acceptable and the unacceptable ( Rubin, 2007). Naming, then, is (also) *assujetissement* ( Foucault, 1994b).

I then went over the specific challenges – in research and in everyday life – of hypernyms, or umbrella terms. In particular, I focused on the existing practices and discourses (academic and not), around so-called “consensual/ethical non-monogamies”. I also noted that part of the politics of naming within neoliberal academia have to do with branding – not with science. To attempt to preemptively address the impacts of this, I outlined a set of publishing procedures and purposeful limitations to the utilization (and utility) of my proposal.

I presented my proposal for an alternative system of classifying both monogamies and non-monogamies for the purposes of research, based on the concept of “explicit”, and explicitness. After delving more into the concept itself, I articulated the definitions, provided examples (and their rationale) and provided suggestions on how to operationalize this contribution, and how it does not necessarily conflict with existing research or is not even intend to fully replace other hypernyms already in use. However, it must be noted that other professionals outside of academia – such as therapists and other mental health practitioners, for example – might also find this classification useful in thinking how to address issues around (non)monogamies, rules, relationship configurations and negotiations and, ultimately, interpersonal communication.

As Gabriel Abend (2019, p. 225) notes, the mobilization of certain concepts – especially thick concepts – requires a series of social conditions for them to even exist in the first place: “[t]hese concepts couldn’t exist if particular social structural, institutional, and cultural facts didn’t obtain”. If the explicitness system contains one particularly thick concept (covert), a series of questions open up as to the plethora of facts that make something like “covert” or “explicit” come into being within the remit of interpersonal relationships, and *as organizing traits* of those relationships. Studying the words people use, but also mobilizing different words to do that, opens up different avenues of research, and allows us to think how different ways of parsing relationships come to be.

My hope for this proposal is not that it be taken-up as-is, but rather that it moves the discussion into a more productive and systematized framework for the academic community. The next step can only be taken through refining and improving it, with the help of other, more experienced, researchers – via transparent, accountable and iterative peer review. It is through this process that newer, improved versions of this classification system can be created, and that critiques to it can be accounted for.

Ultimately, this will, I hope, result in research practices that are more cognizant of their own impact, and that take the politics of naming seriously – and use that naming to break with convention, with systemic norm policing, and with stigma.
