# Supplementary material for: Reframing the role of communication in consensual and/or ethical (non)monogamies: A proposal for a change in academic terminology
Source: Open Res Eur. 2024 Aug 5;4:167. [Version 1] doi: 10.12688/openreseurope.17502.1 (PMC11399770; doi:10.12688/openreseurope.17502.1)
Supplement: Peer review history

# Reviewer report: Reviewer response for version 1

**DOI:** 10.21956/openreseurope.18916.r44087

**Published:** 2024-09-25

**Authors:** Christine Campbell

**Affiliations:** 1 St Mary's University Twickenham, Twickenham, England, UK

I think this is a well-written and engaging article with a thoughtful and intriguing proposal at its heart.

 I have no major critique of the central argument - I think it's a useful contribution to the literature. I like the fact that the author identifies that explicitness is *actually *the point. It's not about ethics or consent, it's about explicitness, very true.

 My only critique is the way the argument is presented. It's a simple proposal. I'd love to see a more simple, shorter paper.

 In terms of balance and organisation of material - it's a slow start. I think some of the argument about naming in general, and Foucault in particular, could be cut and get straight to the point quicker. I actually think that the paragraph about the Ritchie and Barker paper is great and should maybe be moved to the start of the paper. It immediately tells the reader why this topic is important, and it centers the discussion on the topic in question. I'd like to see consensual non-monogamy mentioned sooner - is what I'm saying.

 Stylistically I would prefer rather less jargon. Just from a couple of paragraphs in the introduction I see words like boundarize, identitary. These are words which could be replaced with simpler vocabulary and would make the paper more accessible. But I appreciate that there is a fashion in the field for this approach, bemoan it though I do.

 I do like the central argument - it's very compelling!

 A couple of small points -

 "It is not for naught that it was called poly *amory* – after all, love is more acceptable than sex, since it is further away from, as noted above, pathology-adjacent terms like ‘hysteria’ (and its emphasis on disciplining women’s bodies and pleasures) and ‘hypersexuality’, both focusing on notions of excess." I don't think that the discussion of hysteria is quite relevant to the - accurate - point the author is making about dissociating polyamory from sex, and linking it to the less stigmatised "love". Could there be a better way of illustrating this point - e.g. studies that have shown that people stigmatise purely sexual relationships more than ones which incorporate romantic love?

 I loved the historical approach - might be worth mentioning the ethical slut at this point?

 I didn't follow the point about "academic work behind naming conventions like “designer relationships” or “novogamy” can have deleterious effects on communities, by constraining terminology, by impacting terminology that might be more readily used in policy-making (in a way that is contrary to what communities wish or strive for), and many other examples." Could that be explained a bit more?

Is the argument persuasive and supported by appropriate evidence?

Yes

Is the work clearly and cogently presented?

Yes

Is the topic of the essay discussed accurately in the context of the current literature?

Yes

Does the essay contribute to the cultural, historical, social understanding of the field?

Yes

Reviewer Expertise:

Consensual non-monogamy, sexualised bodies

I confirm that I have read this submission and believe that I have an appropriate level of expertise to confirm that it is of an acceptable scientific standard.

# Reviewer report: Reviewer response for version 1

**DOI:** 10.21956/openreseurope.18916.r44090

**Published:** 2024-09-24

**Authors:** Maria Pallotta-Chiarolli

**Affiliations:** 1 School of Communication and Creative Arts, Deakin University, Burwood, Victoria, Australia

This article provides a thought-provoking and innovative engagement with the politics, marketing and impact of naming, using non-monogamy as the case study. 

 An evolving field of research and advocacy, non-monogamy's terminology, descriptors and boundary-markers increase and change as well. And, as the author demonstrates, naming can be useful in the expansion of  identification and recognition while simultaneously risking the formation of rigid demarcations and new power hierarchies.

 What is also important is the article's discussion of how academia, particularly in its neoliberal status/marketing performances, creates, curates and manipulates naming for its own benefit as well as providing tools for relational living.

 After this critical debate about naming as both opening and oppressing possibility, the author then proposes a new system of umbrella  terms that may be more productive in addressing power, stigma and interpersonal mis/communication. However, in line with the prior discussion, the proposal itself is not presented as expert and conclusive, but rather as moving the discussion forward in ways that invite creative rather than competitive critique.

Is the argument persuasive and supported by appropriate evidence?

Yes

Is the work clearly and cogently presented?

Yes

Is the topic of the essay discussed accurately in the context of the current literature?

Yes

Does the essay contribute to the cultural, historical, social understanding of the field?

Yes

Reviewer Expertise:

Genders, sexualities, relationship and family diversities, cultural diversity, decoloniality.

I confirm that I have read this submission and believe that I have an appropriate level of expertise to confirm that it is of an acceptable scientific standard.

# Reviewer report: Reviewer response for version 1

**DOI:** 10.21956/openreseurope.18916.r44086

**Published:** 2024-09-13

**Authors:** Sharon M. Flicker

**Affiliations:** 1 California State University, Sacramento, USA

I would like to preface this review by providing the context that I am primarily trained as a quantitative researcher and clinical psychologist and my expertise does not extend to this type of paper. Nonetheless, I spend a great deal of time thinking about CNM/ENM and am happy to read this intellectually stimulating paper and offer my thoughts.

*‘Reframing the role of communication in consensual and/or ethical (non)monogamies: A proposal for a change in academic terminology’* highlights the way in which language can be a response to, as well as contribute to, stigma. Thus, the language we use carries great power. The author raises several important issues, making explicit some considerations that have often gone unspoken. For instance, there a great deal of discussion in the literature about how vital communication is to ‘successful’ CNM/ENM but the whats and hows of that communication is not often explicated. The author raises some important questions to be asked by researchers in the Applicability section of the paper to help address this lack.

 The author states ‘ *In this case, I merely wish to engage with a technical-theoretical difficulty: how to use umbrella terms, and which umbrella terms to use, when doing research on relationship types and practices that sit outside the mononormative system*.’ While the term consensual has been debated in the literature and in communities regarding non-monogamy, the lack of discussion around consent is notably missing with regards to monogamy. I would suggest that the concept of explicit and implicit may in fact be even more important in relation to presumed monogamy. Many individuals have strict relationship scripts about monogamy that they assume are shared by others, negating the motivation for conversations as simple as ‘do we want to be monogamous?’ Assuming a couple has had that conversation explicitly, they may not further dive into ‘what constitutes monogamy for us?’; ‘what constitutes acceptable and unacceptable behavior with individuals outside of our dyad?’ Including this dimension of explicitness in research with presumed monogamous samples could be clarifying and rather enlightening. Thus, I believe the suggestions for data collection included in the paper go beyond the limited scope that the author puts forth.

 I offer two thoughts in the spirit of provoking extra thought or explanation:

  

- I was surprised by the use of the term ‘careless’ under the definition of implicit. The term careless has such a negative connotation and conveys (to me at least) an active, rather than a passive, lack of consideration to others. If an individuals’ relationships scripts are so strong that there appear to be no alternatives (to monogamy, pologamy, etc), they may assume that the person with whom they are in a relationship shares the same script. Would this be considered careless if they do not have an understanding that other alternatives exist?
- While explicitness is core to true consent in that people cannot consent if they do not understand what they are consenting to, explicit communication is separate from consent: explicit non-consensual non-monogamy does occur. I would love to see the author address explicit but non-consensual non-monogamy, such as in situations in which one person wields no power to influence the other. While I cannot offer any suggestions for how to avoid the sense of morality that the author suggests is conveyed by this term, consent does seem like a vital dimension that is not fully addressed by the terms proposed in the paper. It would seem that both explicitness and consent are vital dimensions to be assessed.

Is the argument persuasive and supported by appropriate evidence?

Yes

Is the work clearly and cogently presented?

Yes

Is the topic of the essay discussed accurately in the context of the current literature?

Yes

Does the essay contribute to the cultural, historical, social understanding of the field?

Yes

Reviewer Expertise:

Consensual non-monogamy, intimate relationships, quantitative research,

I confirm that I have read this submission and believe that I have an appropriate level of expertise to confirm that it is of an acceptable scientific standard.

# Response

**Published:** 2024-09-13

**Authors:** Daniel Cardoso

**Affiliations:** ECATI, Lusófona University of Humanities and Technologies, Lisbon, Lisbon, Portugal

I would like to thank the work and engagement of your review report, as well as all the work you have done throughout the years on this topic, Both your specific observations are extremely valuable and insightful:

- The use of "careless" was, perhaps in a way that was not fully transparent, inspired by an ethics of care that would, I would argue, place some responsibility on actively attempting to not rely on existing norms or assumptions (though it is, of course, impossible to fully do so); at the same time, and as you rightly say, it is a loaded word that stands at odds with the purpose and driving narrative of the essay itself. I will reflect on this and endeavour to provide a more cogent and theoretically grounded alternative to this term.
- I have raised the possibility (Example 3) of implicit non-consensual monogamy, but I did not delve into it at length and it is equally true that I did not address a case of explicit non-consensual non-monogamy (even though Example 2, i.e., unicorn hunting, might, for some, classify as such). On the next revision of this paper, I will focus on this aspect more specifically - thank you for the challenge!

I would like to add a short clarification - I do not see issues of explicitness as replacing issues of consent (for all the reaons you correctly pointed out); I do see it, as you so well noted, as two connected but distinct aspects that need to be considered in tandem but also in their respective specificities. However, using "consensual" as a classificatory hypernym (rather than as a dimension which can be inquired in any relationship) seems to be, as I have tried to argue, needlessly problematic for scholars and academics working as such. In therapy settings, in social services, and in many other instances, considerations of *practices* of consent are paramount and irrepleaceable, and explicitness is but a supporting element in those considerations.

# Reviewer report: Reviewer response for version 1

**DOI:** 10.21956/openreseurope.18916.r43507

**Published:** 2024-09-09

**Authors:** Christopher Smith

**Affiliations:** 1 General Studies, South University - West Palm Beach (Ringgold ID: 52591), Royal Palm Beach, Florida, USA

This article provides a thorough breakdown of the moral nuances and valuation that impact naming conventions and the need to provide non moralistic specificity to the classification of relationship structures. The paper begins with identifying the how and why non monogamous structures have been classified as ethical or unethical, consensual, or un-consensual and the binary issues that are raised with these paradigms. The author explanation and argument to leave these binary definitions and instead use explicit, implicit or covert as describers of non-monogamous relationship structures provides a clearer and more adaptable paradigm. The tri-paradigm allows for academics and lay to diagnose the way in which communication and clarity are fostered within non monogamous structures and the impact of the extent to which it aids in the understanding relationship dynamics. The author continues on to briefly acknowledge the reality the subjectivity, objectivity and moralistic considerations will always impact the understanding and application of relationship definitions. However, clearly argues that particularly explicitness provides a direction through which people can define a relationship that allows all acting members to maintain a sense of power, choice and self-direction. Knowledge, self-determination and consent thrive in explicitness which ultimately can allow for more clear relationships

Is the argument persuasive and supported by appropriate evidence?

Yes

Is the work clearly and cogently presented?

Yes

Is the topic of the essay discussed accurately in the context of the current literature?

Yes

Does the essay contribute to the cultural, historical, social understanding of the field?

Yes

Reviewer Expertise:

Relationship Structures, Education, Religion, Psychology and Sociology

I confirm that I have read this submission and believe that I have an appropriate level of expertise to confirm that it is of an acceptable scientific standard.
